# Perioperative hypoxemia is common with horizontal positioning during general anesthesia and is associated with major adverse outcomes: a retrospective study of consecutive patients

**DOI:** 10.1186/1471-2253-14-43

**Published:** 2014-06-09

**Authors:** C Michael Dunham, Barbara M Hileman, Amy E Hutchinson, Elisha A Chance, Gregory S Huang

**Affiliations:** 1 Trauma/Critical Services, St. Elizabeth Health Center, 1044 Belmont Avenue, Youngstown OH 44501, USA; 2 Department of Anesthesiology, St. Elizabeth Health Center, 1044 Belmont Avenue, Youngstown OH 44501, USA

**Keywords:** Aspiration, Respiratory, Hypoxemia, Period, Perioperative, Operating rooms, Supine position

## Abstract

**Background:**

Reported perioperative pulmonary aspiration (POPA) rates have substantial variation. Perioperative hypoxemia (POH), a manifestation of POPA, has been infrequently studied beyond the PACU, for patients undergoing a diverse array of surgical procedures.

**Methods:**

Consecutive adult patients with ASA I-IV and pre-operative pulmonary stability who underwent a surgical procedure requiring general anesthesia were investigated. Using pulse oximetry, POH was documented in the operating room and during the 48 hours following PACU discharge. POPA was the presence of an acute pulmonary infiltrate with POH.

**Results:**

The 500 consecutive, eligible patients had operative body-positions of prone 13%, decubitus 8%, sitting 1%, and supine/lithotomy 78%, with standard practice of horizontal recumbency. POH was found in 150 (30%) patients. Post-operative stay with POH was 3.7 ± 4.7 days and without POH was 1.7 ± 2.3 days (p < 0.0001). POH rate varied from 14% to 58% among 11 of 12 operative procedure-categories. Conditions independently associated with POH (p < 0.05) were acute trauma, BMI, ASA level, glycopyrrolate administration, and duration of surgery. POPA occurred in 24 (4.8%) patients with higher mortality (8.3%), when compared to no POPA (0.2%; p = 0.0065). Post-operative stay was greater with POPA (7.7 ± 5.7 days), when compared to no POPA (2.0 ± 2.9 days; p = 0.0001). Conditions independently associated with POPA (p < 0.05) were cranial procedure, ASA level, and duration of surgery. POPA, acute trauma, duration of surgery, and inability to extubate in the OR were independently associated with post-operative stay (p < 0.05). POH, gastric dysmotility, acute trauma, cranial procedure, emergency procedure, and duration of surgery had independent correlations with post-operative length of stay (p < 0.05).

**Conclusions:**

Adult surgical patients undergoing general anesthesia with horizontal recumbency have substantial POH and POPA rates. Hospital mortality was greater with POPA and post-operative stay was increased for POH and POPA. POH rates were noteworthy for virtually all categories of operative procedures and POH and POPA were independent predictors of post-operative length of stay. A study is needed to determine if modest reverse-Trendelenburg positioning during general anesthesia has a relationship with reduced POH and POPA rates.

## Background

Perioperative pulmonary aspiration (POPA) can cause death [1-4] and may lead to clinically significant morbidities [1,4,5]. It is important to note that reliable estimates of POPA rates are uncertain, in part, due to a lack of prospective data. Adult POPA rates from retrospective comprehensive database reviews have ranged from 0.01% to 0.9% [4,6-11], while rates from voluntary claims reporting databases have varied from 1.4% to 2.9% [5,12-14]. Besides variability in reported POPA rates, another concern has been the ability to determine, with precision, when pulmonary aspiration has or has not occurred. Clinical certainty is evident when there is aspiration of bile or particulate matter from the tracheobronchial tree or there is endoscopic visualization of aspirated material [10,11,13,15,16]. However, the diagnosis is more presumptive when there is development of a new intra-operative or post-operative infiltrate seen on a chest x-ray and attendant tachypnea, hypoxia, wheezing, or changes in ventilator airway pressures [10,11,13,15,16].

There is substantial operating room, intensive care unit (ICU), and animal investigative evidence that aspiration occurs despite the presence of a cuffed endotracheal tube [17-22]. In addition, multiple pre-operative host clinical conditions may increase the risk for POPA; however, precise probabilities are uncertain. Such conditions include solid or non-clear liquid consumption within six hours of surgery, bowel obstruction, ileus, acute abdomen, morbid obesity, diabetic gastroenteropathy, gastroesophageal reflux disease, hiatal hernia, active peptic ulcer disease, pre-operative opioids, ascites, advanced pregnancy, large abdominal tumor, large abdominal organomegaly, acute trauma, and alcohol intoxication [9,23-29]. Because these conditions are not unusual in operative patients, vigilant clinical concern for the development of POPA has been advocated [16,22,24,30].

Extensive clinical evidence from the literature demonstrates that the horizontal positioning in mechanically ventilated patients is a risk for pulmonary aspiration with lung inflammation [22,31] and ventilator-associated pneumonia [17,18,32-37]. Accordingly, the Institute for Healthcare Improvement recommends elevating the head of the bed to prevent pulmonary aspiration and ventilator-associated pneumonia, during ICU mechanical ventilation [38]. Patients undergoing general endotracheal anesthesia for a surgical procedure are primarily placed in a supine, lithotomy, lateral, or prone position [6,39,40], where horizontal recumbency is typically enforced [39-41]. It seems logical that horizontal recumbency, as a common practice, is counterintuitive, when considering literature evidence regarding risks for POPA.

For these reasons, the current investigation was designed to determine the rate of POPA in surgical patients undergoing endotracheal intubation, general anesthesia, and a diverse array of procedures. Because hypoxemia is a common manifestation with pulmonary aspiration [42-44] and pulse oximetry monitoring is a routine practice, we used perioperative hypoxemia (POH) as a potential signal for POPA. We assessed each surgical patient during the operative procedure and the subsequent 48 hours for POH. Patients were categorized as encountering POPA, if they had POH and post-operative radiographic imaging (chest x-ray or CT scan) demonstrating an acute pulmonary infiltrate. Of interest, we found only one investigation of POH in a group of patients undergoing a diverse array of surgical procedures, following Post Anesthesia Care Unit (PACU) discharge [45]. We hypothesized that patients with POH and the subset cohort with POPA (POH with pulmonary infiltrate) would each have a clinically substantial occurrence rate. We also conjectured that patients with POH and the sub-group with POPA would have increased adverse clinical outcomes.

## Methods

This Humility of Mary Health Partners Institutional Review Board approved study was a retrospective review of 500 consecutive patients aged 18 years or older, had pre-operative pulmonary stability, and underwent an operative procedure that required endotracheal intubation and a general anesthetic. Patients were identified through the surgery case log, and the data were collected from the electronic medical record (EMR). Consequently, a patient consent form was waivered by the Institutional Review Board. Exclusion criteria were tracheal intubation prior to emergency department arrival, thoracotomy procedure, any cardiac procedure, Glasgow Coma Score < 13, an American Society of Anesthesiology (ASA) classification of V or VI, and patients with more than one surgery requiring tracheal intubation during the same hospitalization. Pre-operative pulmonary stability criteria was defined as a respiratory rate 12–24 breaths per minute and either a SpO_2_ ≥ 94% when breathing room air or receiving nasal cannula oxygen with a flow rate 1to 2 liters per minute or PaO_2_/FiO_2_ ≥ 300, if receiving greater supplemental oxygen.

### Host conditions

The following pre-existing host conditions were documented in the data base: (1) age, (2) gender, (3) esophagogastric dysfunction, (4) gastric dysmotility, (5) intestinal dysmotility, (6) abdominal hypertension, (7) recent eating, (8) pre-existing lung condition, (9) acute trauma, (10) weight, and (11) body mass index (BMI). Esophagogastric dysfunction was defined as the presence of gastroesophageal reflux or hiatal hernia.

Gastric dysmotility was defined as the presence of active peptic ulcer disease, vomiting within eight hours of surgery, upper gastrointestinal bleeding within eight hours of surgery, or intravenous narcotic administration within four hours of surgery. Intestinal dysmotility was defined as the presence of bowel obstruction, ileus, or an acute abdominal condition. Abdominal hypertension was define as the presence of morbid obesity (BMI ≥ 40), ascites, increased abdominal girth, pregnancy > 12 weeks, large abdominal tumor, or large abdominal organomegaly. Pre-operative eating was defined as the consumption of solid food or non-clear liquids within six hours of surgery. A pre-existing lung condition was considered present when a patient required daily home bi-level positive airway pressure, supplemental oxygen, inhalational bronchodilator, or systemic bronchodilator or steroid. Acute trauma was defined as any injury occurring within 24 hours prior to admission. The above information was ascertained by reviewing the anesthesia pre-operative assessment note and the history and physical examination documented in each patient’s EMR.

### Operative conditions

Specific operative procedures were classified into one of the following 11 categories: cranial, facial soft tissue, intra-oral, laparotomy, laparoscopy, spinal, neck (non-spinal), breast, extremity/pelvis, aortic, and miscellaneous. The operative body position was documented as prone, decubitus, sitting, or supine or lithotomy as indicated on the anesthesia intra-operative record. Standard anesthesia practice was to maintain horizontal recumbency, except for patients in the sitting position. The following data were gathered from the anesthesiology intra-operative record: the use of the Trendelenburg position, ASA classification level along with emergency status, the utilization of rapid-sequence induction and cricoid pressure, duration of surgery in minutes, fluid intake, fluid output, and administration of intravenous glycopyrrolate with anesthesia induction.

### Patient outcomes

Hospital mortality status, total hospital length of stay, and the post-operative duration of hospitalization were obtained from the EMR. For patients discharged < 36 hours after surgery, institutional policy requires telephone contact be attempted for patient follow-up. If contact was made, notation as to whether or not the patient had any substantial post-operative problem was documented. Additionally, for the patients discharged the same day as surgery or the day following surgery, the EMR, which includes the hospital corporations’ three area hospitals, was interrogated for emergency department visits and hospital readmission. All patient contact with a corporate emergency department, hospital, or clinic was reviewed to determine whether evidence for pulmonary insufficiency existed. This follow-up assessment was undertaken as an attempt to provide a more comprehensive appraisal of patients undergoing early post-operative discharge.

### Hypoxemia outcomes

Because perioperative pulse oximetry monitoring is a routine at our institution, we used POH as a potential signal for POPA. A co-investigator examined each patient’s anesthesia operative record and documented the presence of intra-operative hypoxemia, when SpO_2_ < 98% was identified. A co-investigator also screened the EMR for evidence of POH. A positive post-operative hypoxemia screen was defined as two or more episodes of SpO_2_ < 94%, on room air or nasal cannula supplemental oxygen at 1–2 liters per minute, or < 98% with greater supplemental oxygen, within a 24-hour period, during the 48 hours following surgery. SpO_2_ < 94% during the first-two hours following operating room extubation were not counted as a post-operative hypoxemic event, as hypoventilation may be related to post-anesthesia recovery. The first author, a board certified surgical intensivist, reviewed each patient’s data whenever a patient had intra-operative hypoxemia and/or a positive screen for post-operative hypoxemia. Whenever the intra-operative SpO_2_ was clearly < 98% and the intra-operative FiO_2_ was subsequently increased, the patient was classified as having an episode of intra-operative hypoxemia. When the post-operative hypoxemia screen was positive, the first-author reviewed each patient’s post-operative pulse oximetry results. When the post-operative SpO_2_ had a ≥ 5% reduction, as compared to their pre-operative value, the patient was categorized as having an episode of post-operative hypoxemia. POH was considered to be present if intra-operative and/or post-operative hypoxemia was documented. Failure to extubate the patient in the operating room was documented in the data base.

### Aspiration outcomes

POPA was defined as the presence of POH and an acute pulmonary infiltrate on thoracic radiographic imagining (chest x-ray or CT scan) within the 48-hour period following surgery. The first-author examined each chest radiographic image (chest x-ray or CT scan) available in the EMR during the 48-hour post-operative period in patients categorized with POH, for a pulmonary infiltrate. When the first-author’s findings of an infiltrate were corroborated by the radiologist’s report, the patient was classified as POPA-positive.

### Statistical analysis

Statistical relationships for POH and POPA with host and operative conditions and post-operative length of hospitalization were performed. Data were entered into a Microsoft Excel® 2010 spreadsheet and imported into a SAS System for Windows, release 9.2 (SAS Institute Inc., Cary, NC, USA), to perform statistical analyses. For continuous variable cohort data, standard deviation was used to complement the mean. Correlation coefficient analysis was used to assess relationships between two continuous data variables. Non-parametric analysis was used to compare continuous data results between two groups. ANOVA was applied to compare continuous data involving more than two groups. Fischer’s exact testing was used to assess the relationship of two dichotomous variables. Multivariate logistic regression analysis was performed to assess independent variable relationships with a dependent variable that was dichotomous. Multivariate regression analysis was applied to evaluate independent variable relationships with a dependent variable that was continuous. A p < 0.05 was considered to represent a statistically significant relationship.

## Results

From May 14 through July 13, 2012, 500 consecutive, eligible patients were investigated. Host conditions are in Table 1.

**Table 1:** Host conditions

**Age (years)**	54.2 ± 17
**Male**	197 (39.4%)
**Female**	303 (60.6%)
**Esophagogastric dysfunction**	170 (34.1%)
**Gastric dysmotility**	54 (10.8%)
**Intestinal dysmotility**	15 (3.0%)
**Abdominal hypertension**	63 (12.6%)
**Eating within 6 hours of surgery**	16 ( 3.2%)
**Pre-existing lung disease**	69 (13.8%)
**Acute trauma**	37 ( 7.4%)
**Pre-operative FiO**_**2**_**:**	
**room air**	425 (85.0%)
**low-flow nasal cannula**	63 (12.6%)
**no documentation**	12 ( 2.4%)
**Pre-operative SpO**_**2 **_**(%)**	97.7 ± 1.9
**Pre-operative respiratory rate (bpm)**	18.1 ± 1.9
**Height (feet)**	5.5 ± 0.4
**Weight (kilograms)**	86.2 ± 24.3

### Operative conditions

The operative procedures are listed in Table 2. The operative body position was prone in 66 (13.2%), decubitus in 38 (7.6%), sitting in 4 (0.8%), and supine or lithotomy in 392 (78.4%). Standard anesthesia practice was to maintain horizontal recumbency, except for the few patients in a sitting position. The Trendelenburg position was utilized in 27 (5.4%) patients. The mean ASA level was 2.8 ± 0.6 (I-IV) with a level of I for 12 (2.4%) patients, II for 129 (25.8%) patients, III for 318 (63.6%) patients, and IV for 41 (8.2%) patients. The ASA status was categorized as emergent in 36 (7.2%) patients, with the remaining 464 (92.8%) considered to have been elective cases. Rapid-sequence induction was performed in 43 (8.6%) patients, and cricoid pressure was applied during induction in 42 (8.4%) patients. During the operative procedure, the duration of anesthesia was 129 ± 77 (18–500) minutes, fluid infusion was 1.8 ± 1.2 liters, and fluid input and output balance was 1.4 ± 1.1 liters. Intravenous glycopyrrolate was administered to 119 (23.8%) patients immediately prior to initiation of the surgical procedure. Patients given glycopyrrolate had higher body weight (p = 0.0204) and were more likely to be placed in the prone position (p < 0.0001).

**Table 2:** Operative procedures

**Procedure**	**Number**	**Percent**
**Cranial**	19	3.8%
**Facial soft tissue**	1	0.2%
**Intra-oral**	28	5.6%
**Laparotomy**	49	9.8%
**Laparoscopy**	103	20.6%
**Spinal**	80	16.0%
**Neck (non-spinal)**	26	5.2%
**Breast**	28	5.6%
**Extremity/pelvis**	112	22.4%
**Aortic**	8	1.6%
**Miscellaneous**	46	9.2%

### Patient outcomes

Of the 500 patients, 19 (3.8%) could not be extubated in the operating room. Only three (0.6%) patients died prior to hospital discharge. The mean total hospital length of stay was 3.3 ± 4.1 days and post-operative duration of hospitalization was 2.3 ± 3.3. The number of days after surgery until hospital discharge was 0 days in 142 (28.4%) patients, 1 day in 139 (27.8%), 2 days in 60 (12.0%), 3 days in 51 (10.2%), 4 days in 33 (6.6%), and ≥ 5 days in 75 (15.0%). For the 162 patients discharged within < 36 hours after surgery, 85 (52.5%) had a telephone conversation, with no patient indicating that they had any substantial post-operative problem. Of the 281 patients discharges the same day as surgery or the day following surgery, 14 (5.0%) were seen in an emergency department or had hospital readmission; however, none had evidence of respiratory insufficiency.

### Hypoxemia outcomes

Intra-operative hypoxemia occurred in 40 (8.0%) patients, while post-operative hypoxemia was noted in 128 (25.6%) patients. POH, intra-operative and/or post-operative, was found in 150 (30.0%) of the 500 patients. For the 150 patients with POH, the number of days from surgery until hospital discharge was greater (3.7 ± 4.7 days), when compared to those without hypoxemia (1.7 ± 2.3 days; p < 0.0001). This represented a two-fold increase in the number of post-operative days, that is, an additional two days of hospitalization per patient with POH. The rate of POH varied from 14.3% to 57.9% among 11 of the 12 operative procedure categories (Table 3). According to body position, the POH rate was prone 28.8%, decubitus 44.7%, sitting 0%, and supine or lithotomy 29.1%. POH was associated with age, abdominal hypertension, weight, BMI, cranial procedures, decubitus position, ASA level of classification, duration of surgery, glycopyrrolate administration, and inability to extubate in the OR (Table 4). The POH rate was lower with glycopyrrolate administration (20.2% [24/119]), when compared to no glycopyrrolate (33.1% [126/381]; p = 0.0082; odd ratio = 2.0). The odds ratio for inability to extubate POH patients in the operating room, when compared to those without POH, was 22.2.

**Table 3:** Perioperative hypoxemia rates by operative procedure

**Procedure**	**Number**	**Hypoxia rate**
**Cranial**	19	57.9%
**Facial soft tissue**	1	0%
**Intra-oral**	28	21.4%
**Open laparotomy**	49	49.0%
**Laparoscopy**	103	22.3%
**Spinal**	80	30.0%
**Neck (non-spinal)**	26	38.5%
**Miscellaneous**	46	15.2%
**Breast**	28	14.3%
**Extremity/pelvis**	112	33.0%
**Aortic**	8	50.0%

**Table 4:** Perioperative hypoxemia associations

	**No hypoxia**	**Hypoxia**	**P-value**
**Number**	350 (70.0%)	150 (30.0%)	
**Fluid input (−) output**	1.3 ± 1.0	1.5 ± 1.2	0.0475
**Fluid input (mL per hour)**	938 ± 470	870 ± 498	0.1483
**OR minutes**	119 ± 70	152 ± 88	< 0.0001
**ASA level**	2.7 ± 0.7	3.0 ± 0.5	< 0.0001
**Age**	52.2 ± 17	59.0 ± 17	< 0.0001
**Pre-existing lung disease**	12.0%	18.0%	0.0747
**Weight (kg)**	84 ± 23	92 ± 27	0.0024
**BMI**	29.5 ± 7.6	32.0 ± 8.4	0.0012
**Glycopyrrolate**	27.1%	16.0%	0.0082
**Acute Trauma**	6.0%	10.7%	0.0677
**Increased IAP**	9.7%	19.3%	0.0030
**Decubitus position**	6.0%	11.3%	0.0392
**Cranial procedure**	2.3%	7.3%	0.0068
**Not extubated in OR**	0.6%	11.3%	< 0.0001

OR: operating room; ASA: American Society of Anesthesiologists; BMI: body mass index; IAP: intra-abdominal pressure.

A trend for a correlation with POH existed for patients with trauma and pre-existing lung disease (Table 4). POH did not correlate with fluid input during surgery, esophagogastric dysfunction, gastric dysmotility, intestinal dysmotility, Trendelenburg position, non-decubitus positioning, non-cranial procedures, emergency procedures, rapid sequence induction, or cricoid pressure (Table 4). Although the mean age of POH patients was slightly higher, it was less than 65 (Table 4). Conditions independently associated with POH were acute trauma (p = 0.0225), BMI (p = 0.0033), glycopyrrolate administration (p = 0.0031), ASA level (p < 0.0001), and duration of surgery (p = 0.0002).

### Aspiration outcomes

Of the 500 patients, 24 (4.8%) met the criteria for definite POPA. Mortality was greater in the patients with POPA (8.3% [2/24]), when compared to the patients without POPA (0.2% [1/476]; p = 0.0065; OR 43.2). For the 24 patients with POPA, the number of days from surgery until hospital discharge was greater (7.7 ± 5.7 days), when compared to those without POPA (2.0 ± 2.9 days; p = 0.0001). The additional post-operative length of stay for the POPA patients represents a nearly four-fold increase. POPA had associations with cranial procedure, prone positioning, ASA level, duration of surgery, failure to extubate in the OR, and prolonged post-operative intubation, (Table 5). POPA did not correlate with age, esophagogastric dysfunction, gastric dysmotility, intestinal dysmotility, abdominal hypertension, acute trauma, weight, BMI, Trendelenburg position, emergency procedures, rapid sequence induction, pre-existing lung disease, cricoid pressure, or fluid input during surgery. Conditions independently associated with POPA were cranial procedures (p = 0.0445), ASA level (p = 0.0209), and duration of surgery (p < 0.0001).

**Table 5:** Conditions associated with pulmonary aspiration

	**No aspiration**	**Aspiration**	**P-value**
**Number of patients**	**476**	**24**	
**ASA level**	2.8 ± 0.6	3.2 ± 0.6	0.0017
**ASA level 4**	3.9%	14.6%	0.0021
**Fluid Input (mL per hour)**	912 ± 458	1,037 ± 802	0.4551
**OR minutes**	125 ± 71	211 ± 127	0.0031
**Not extubated in OR**	1.9%	41.7%	< 0.0001
**Post-op intubation > 24 hrs.**	0.2%	20.8%	< 0.0001
**Cranial procedure**	3.4%	12.5%	0.0223
**Decubitus position**	4.1%	13.2%	0.0122

ASA: American Society of Anesthesiologists; OR: operating room.

### Post-operative length of stay

The post-operative length of stay, in days, had associations with POPA, POH, age, gastric dysmotility, acute trauma, cranial procedure, non-supine/lithotomy positioning, ASA level, emergency procedures, rapid sequence induction, cricoid pressure, duration of surgery, and inability to extubate in the OR (Table 6). The post-operative length of stay did not correlate with esophagogastric dysfunction, intestinal dysmotility, abdominal hypertension, pre-existing lung disease, weight, BMI, Trendelenburg position, or fluid input during surgery. Conditions independently associated with post-operative length of stay were POPA (p < 0.0001), acute trauma (p < 0.0001), duration of surgery (p < 0.0001), and inability to extubate in the OR (p = 0.0077). Circumstances shown to have an independent correlation with post-operative length of stay were POH (p < 0.0001), gastric dysmotility (p = 0.0006), acute trauma (p = 0.0027), cranial procedure (p < 0.0001), emergency procedure (p = 0.0017), and duration of surgery (p < 0.0001).

**Table 6:** Conditions associated with post-operative length of stay (days)

**Conditions**	**Conditions**	**P-value**
**No aspiration**	**Aspiration**	
2.0 ± 2.9	7.7 ± 5.9	< 0.0001
**No hypoxemia**	**Hypoxemia**	
1.7 ± 2.3	3.7 ± 4.7	< 0.0001
**Elective**	**Emergency**	
2.1 ± 3.1	4.7 ± 4.6	0.0017
**Non-trauma**	**Acute trauma**	
2.1 ± 2.9	4.9 ± 5.9	0.0072
**Extubate in OR**	**Not extubate in OR**	
2.1 ± 2.9	8.0 ± 6.8	0.0014
**Non-cranial procedure**	**Cranial procedure**	
2.2 ± 3.2	6.0 ± 4.0	< 0.0001
**No gastric dysmotility**	**Gastric dysmotility**	
2.1 ± 3.2	3.9 ± 4.1	0.0023
**No rapid sequence induction**	**Rapid sequence induction**	
2.2 ± 3.3	3.5 ± 3.5	0.0136
**No cricoid pressure**	**Cricoid pressure**	
2.2 ± 3.3	3.5 ± 3.5	0.0182
**Non-supine/lithotomy position**	**Supine/lithotomy position**	
3.1 ± 4.2	2.1 ± 3.0	0.0189
	**Age**	0.0001
	**ASA level**	0.0006
	**Duration of surgery**	< 0.0001

OR: operating room; ASA: American Society of Anesthesiologists.

## Discussion

### Perioperative hypoxemia outcomes

The POH in the current study was found to be 30.0%. Ehrenfeld et al. demonstrated an intra-operative hypoxemia rate of 6.8% [46], while several other studies have documented PACU hypoxemia rates ranging from 17% to 50% [47-52]. Several investigators have found substantial POH during the first few days following abdominal surgery [53-55] or hip fracture surgery [56]. Lampe et al. published the only study that monitored pulse oximetry following discharge from the PACU in a group of patients undergoing diverse operative procedures [45]. During the first 24 hours following surgery, POH occurred in 60% of patients, with oxygen saturation improving on post-operative day two. Similar to our study, the literature corroborates the observation that post-operative POH is a frequent entity.

It is likely that the POH rate in the current study would have been higher had we not excluded hypoxemic events occurring during the first two hours following surgery. Investigators have described high rates of POH during the first post-operative hour in the PACU; however, they radically decrease over the subsequent one-hour [47,52]. For the patients with POH, the number of days from surgery until hospital discharge was greater, when compared to those without POH. The two-day increased hospitalization for the 150 hypoxemic patients represents 300 days for the two months, which extrapolates to 1,800 additional hospital days for the year. Of importance, POH had an independent correlation with post-operative length of stay.

The rate of POH was substantial in virtually all of the 12 operative procedure categories. Although the primary operative body position was supine or lithotomy, the standard anesthesia practice was to maintain horizontal recumbency in all patients, except for the few patients in the sitting position. POH was associated with age, abdominal hypertension, weight, BMI, cranial procedures, decubitus position, ASA level, duration of surgery, and inability to perform extubation in the OR. Perioperative hypoxemic patients were older; however, the average remained less than 65, indicating that they were not elderly. According to the literature, PACU POH has been associated with the following similar conditions: increasing age [47], obesity [49,50], ASA level [48,49], and duration of surgery [48,49]. The association of abdominal hypertension with POH in the current study may represent a mechanical effect, similar to weight, BMI, and obesity. The reasons for increased POH with the decubitus position and cranial procedures are uncertain. Conditions independently associated with POH in the current study were acute trauma, BMI, cranial procedures, ASA level, and duration of surgery. Lampe et al. found that post-operative oxygen saturation values were lower with older patients; however, age did not significantly increase the rate of POH in the post-operative period [45].

### Perioperative hypoxia mechanism

To try to understand the potential mechanistic foundation for POH in the current study is intriguing. The analysis indicates that intra-operative fluid excess, elderly-age, and pre-existing lung disease were not POH risk factors. However, POH was associated with older age, abdominal hypertension, acute trauma, weight, BMI, cranial procedures, decubitus position, ASA level, duration of surgery, and glycopyrrolate administration. These observations suggest that conditions other than pulmonary edema or obstructive-restrictive lung disease were principals.

We found that glycopyrrolate administration was an independent predictor of POH. Parenteral glycopyrrolate has been shown to decrease oral, tracheobronchial, and gastric secretions [57-60]. Although the precise reasons for administering intravenous glycopyrrolate in the current study are unclear, administration is a discretionary decision [61] and is typically considered when it is important to decrease secretory production or prevent bradycardia [62]. The lower POH rate with glycopyrrolate is mechanistically consistent with the notion that pulmonary aspiration may have been a factor in patients developing POH. The lower POH rate with glycopyrrolate establishes an additional link, along with duration of surgery, decubitus positioning, and cranial procedures, between POH and events that transpired during the operative procedure. Further, the multiple intra-operative conditions associated with POH (duration of surgery, glycopyrrolate administration, cranial procedures, and decubitus position) and the increased rate of inability to extubate POH patients in the operating room suggests that POH pulmonary injury was related to intra-operative events.

Some of the conditions associated with POH in the current study have also been linked to POPA or regurgitation and include the following: increased age [4,9,22], acute trauma [24,31], obesity [9,22,24,30], increased ASA level [11,22,30], and increased duration of surgery [6,30]. In the current study, the rate of POH for open laparotomy was 49% and abdominal hypertension was found to have an association with POH. Some experts have found evidence that abdominal pathology and procedures increase the risk for POPA [22].

Just as POH was found to be a ubiquitous event in the current study, Blitt et al. found compelling evidence, in a prospective study, that regurgitation occurred in all surgical body positions [6]. Other researchers have also found pervasive presence of POPA among the multiple types of surgery that were investigated in each of four studies [4,8,9,11]. The current study findings and literature documentation are consistent with the notion that POH, in part, may be a manifestation of occult- or micro-pulmonary aspiration during horizontal recumbency.

### Perioperative pulmonary aspiration outcomes

Published POPA rates are higher (1.4% to 2.9%) for investigations from voluntary claims reporting databases [5,12-14], when compared to studies emanating from comprehensive database reviews (0.01% to 0.9%) [4,6-11]. The nearly 5% POPA rate in the current study is higher than any published rate, a finding especially noteworthy when considering that our investigation is functionally a comprehensive database review. The seven historic comprehensive database reviews include the intra-operative and early post-operative periods in three studies [8,10,11], the intra-operative period only in three investigations [6,7,9], and an unspecified time period in one study [4]. Data emanates from an anesthesia database in five of the investigations [7-11], a prospective database in one [6], and a statewide surgical database in another [4].

In the seven comprehensive database studies, the traits for determining POPA included non-respiratory secretions in four investigations [7,8,10,11] and post-operative chest x-ray infiltrates in two studies [10,11]. The investigation by Kozlow et al. required a discharge diagnosis of aspiration pneumonia within a statewide surgical database [4]. The study by Olsson et al. did not specify the characteristics for POPA, only that it be documented in the anesthesia database [9]. The Blitt et al. research was prospective and was an active search for regurgitation and aspiration [6].

The higher POPA rate in the current study is likely related to our reliance on POH monitoring as a signal for potential POPA and extending the period of observation to the first 48 post-operative hours. Ideally, all patients would have had a pre-operative and post-operative chest x-ray to detect a new perioperative infiltrate. This might have revealed a similar, higher, or lower POPA rate compared to the current study results. A requirement for pre-operative and post-operative radiographs in all patients would create operational complexity, e.g., funding for the investigation. Although one may quibble with our methodology, the fact that POPA patients had a higher mortality and substantially long hospitalization following surgery provides credibility.

Mortality was greater in the patients with POPA, when compared to the patients without POPA. Historic data documented in five publications provides evidence that POPA mortality rates have ranged from 1.5% to 15.6% [5,9,11,14,63]. Further, Kozlow et al. showed that POPA mortality was increased with an odds ratio of 7.6, when compared to patient mortality without POPA [4].

In the current study, the number of days from surgery until hospital discharge had nearly a four-fold increase in POPA patients, when compared to those without POPA. Importantly, POPA was independently associated with post-operative length of stay, along with duration of surgery and an acute traumatic condition. The study by Kozlow et al. demonstrated that surgical patients with aspiration pneumonia had a total hospital stay of nine days longer, in comparison to the non-POPA group [4]. Of relevance, investigators have demonstrated that admission to an ICU has been warranted in 27% to 57% of patients with POPA [10,11,14].

In the current study, POPA had associations with cranial procedure, decubitus positioning, ASA level, duration of surgery, failure to extubate in the OR, and prolonged post-operative intubation. Of relevance, the proactive investigation by Blitt et al., demonstrated that nine percent of patients under general anesthesia were demonstrated to have regurgitated [6] and Kluger et al. showed that 55% of patients with vomiting or regurgitation had pulmonary aspiration [5]. The Blitt study also proved that regurgitation was significantly more likely when the duration of the operative procedure was > two hours [6]. The Blitt investigation further showed that regurgitation occurred in 8% with decubitus positioning and 17% of neurosurgical procedures [6]. The increased rates of inability to extubate POPA patients in the operating room and prolonged post-operative intubation, in the current study, suggest that the pulmonary inflammatory process was related to the surgical procedure. Increased ASA levels have also been documented in the literature to be associated with higher rates of pulmonary complications [11] and POPA [10].

### Horizontal recumbency

Substantial evidence from the literature indicates that horizontal recumbency during mechanical ventilation creates a risk for pulmonary aspiration with lung injury [22,31] or ventilator-associated pneumonia [17,18,32-37]. The supine, lithotomy, prone, decubitus, and sitting positions are considered to be the most common anatomic postures utilized during surgical procedures [6,39,40]. In the current study, the primary operative body position was supine or lithotomy, a finding analogous to that of Blitt et al. [6]. In the current study, standard anesthesia practice was to maintain horizontal recumbency, except for the few patients in the sitting position. Horizontal recumbency, for the typical operative body positions, is promulgated within the operative nursing literature and teaching circles, as common practice [39-41]. Specifically, horizontal positioning is disseminated by the use of specific narrative description statements [39,40] and inclusion of illustrations and photographs [39,41,64] that portray horizontal recumbency.

We evaluated four review publications, related to POPA, for comments regarding body positioning. The most current review includes only a single comment regarding body positioning [30] and another makes no mention of body positioning [16]. Ng et al. indicate that the Trendelenburg position is a risk for POPA and lithotomy positioning may be a risk [24]. The greatest attention to body position, as a risk for POPA, was in a review publication by Kalinowski et al. in 2004 [22]. Relevant statements in the manuscript indicate that aspiration is common in patients with impaired consciousness in the supine position and with successful tracheal intubation, pulmonary aspiration appears to be less frequent if the head is elevated 45 degrees [22].

In the current study, POH was a common occurrence among the various intra-operative body position postures and the multiple surgical procedural categories. Because POH and horizontal recumbency were pervasive in the current study, it is compelling to consider that these two conditions may be linked. We believe the multiple findings in the current study and the literature link horizontal recumbency to POPA and POH.

### Study limitations

Routine pre-operative and post-operative radiographic chest imaging would have been ideal. Clear lung fields on the pre-operative film would have provided greater evidence that each patient had pre-surgical pulmonary stability. However, the pre-operative SpO_2_ and respiratory rate values are convincing. Routine post-operative chest imaging would have provided a more accurate determination for pulmonary inflammation in patients with or without POH. Thus, the rate of POPA would have been more precise. However, the POPA rate would have only increased, because we did not categorize any patient with POPA, unless a concomitant chest radiographic image demonstrated a pulmonary infiltrate.

## Conclusions

Even though procedures were primarily elective, adult surgical patients undergoing general anesthesia had substantial POH and POPA rates with horizontal recumbency, despite endotracheal intubation. Hospital mortality was greater with POPA and post-operative lengths of stay were increased for POH and POPA patients. POH rates were noteworthy for virtually all categories of operative procedures and body position postures. POH was independently associated with pre-existing host complications, acute trauma, body size, cranial procedures, and length of the surgical procedure. Conditions independently associated with POPA were pre-operative patient complexity and duration of surgery. POH and POPA were shown to be independent predictors of post-operative length of stay. The current study findings and literature documentation are consistent with the notion that POH, in part, may be a manifestation of occult- or micro-pulmonary aspiration during horizontal recumbency. Future studies may show that modest reverse Trendelenburg positioning during general anesthesia is associated with lower POH and POPA rates.

## Abbreviations

ASA: American society of anesthesiology; BMI: Body mass index; EMR: Electronic medical record; ICU: Intensive care unit; PACU: Post anesthesia care unit; POH: Perioperative hypoxemia; POPA: Perioperative pulmonary aspiration.

## Competing interests

The authors declared that they have no competing interests.

## Authors’ contributions

CMD, BMH, AEH, EAC, and GSH conceptualized and designed the study. CMD, BMH, and , EAC were involved in the day-to-day oversight of the study. CMD, BMH, and EAC performed the data collection. CMD performed the data analysis. CMD, BMH, AEH, EAC, and GSH performed the data interpretation. CMD, BMH, EAC, and GSH performed the literature search and drafted the manuscript. CMD, BMH, AEH, EAC, and GSH critically revised the manuscript for important intellectual content. All authors made substantial contributions to conception and design, or acquisition of data, or analysis and interpretation of data. All authors have been involved in drafting the manuscript or revising it critically for important intellectual content. All authors read and approved the final manuscript.

## Authors’ information

CMD has 35 years experience as a Trauma Surgeon and is a board certified Surgical Intensivist and is a board certified General Surgeon. BMH and EAC are experienced full-time research assistants for The Trauma and Orthopedics Research Department. AEH is a board certified Anesthesiologist and the Chief of Anesthesiology. GSH is a board certified General Surgeon, a Trauma Surgeon, and a board certified Surgical Intensivist.

## Pre-publication history

The pre-publication history for this paper can be accessed here:

http://www.biomedcentral.com/1471-2253/14/43/prepub
